# Non-synonymous Substitutions in HIV-1 GAG Are Frequent in Epitopes Outside the Functionally Conserved Regions and Associated With Subtype Differences

**DOI:** 10.3389/fmicb.2020.615721

**Published:** 2021-01-11

**Authors:** Babatunde A. Olusola, David O. Olaleye, Georgina N. Odaibo

**Affiliations:** Department of Virology, College of Medicine, University of Ibadan, Ibadan, Nigeria

**Keywords:** non-synonymous substitutions, HIV-1 GAG, MHR, HIV-1 subtypes, HIV cure

## Abstract

In 2019, 38 million people lived with HIV-1 infection resulting in 690,000 deaths. Over 50% of this infection and its associated deaths occurred in Sub-Saharan Africa. The West African region is a known hotspot of the HIV-1 epidemic. There is a need to develop an HIV-1 vaccine if the HIV epidemic would be effectively controlled. Few protective cytotoxic T Lymphocytes (CTL) epitopes within the HIV-1 GAG (HIV_gagconsv) have been previously identified to be functionally conserved among the HIV-1 M group. These epitopes are currently the focus of universal HIV-1 T cell-based vaccine studies. However, these epitopes’ phenotypic and genetic properties have not been observed in natural settings for HIV-1 strains circulating in the West African region. This information is critical as the usefulness of universal HIV-1 vaccines in the West African region depends on these epitopes’ occurrence in strains circulating in the area. This study describes non-synonymous substitutions within and without HIV_gagconsv genes isolated from 10 infected Nigerians at the early stages of HIV-1 infection. Furthermore, we analyzed these substitutions longitudinally in five infected individuals from the early stages of infection till after seroconversion. We identified three non-synonymous substitutions within HIV_gagconsv genes isolated from early HIV infected individuals. Fourteen and nineteen mutations outside the HIV_gagconsv were observed before and after seroconversion, respectively, while we found four mutations within the HIV_gagconsv. These substitutions include previously mapped CTL epitope immune escape mutants. CTL immune pressure likely leaves different footprints on HIV-1 GAG epitopes within and outside the HIV_gagconsv. This information is crucial for universal HIV-1 vaccine designs for use in the West African region.

## Introduction

Thirty-eight million people lived with HIV-1 infection in 2019, with 690,000 AIDS-related deaths. Over 50% of HIV infection and about 63% of its associated deaths occurred in Sub-Saharan Africa (UNAIDS, 2020). The rate of new infections also increased in the region despite a 40% global reduction in new infections since 1998 (UNAIDS, 2020; WHO, 2020). About 69% of these new HIV infections occurred in Western and Central Africa. Nigeria accounts for over 60% of new HIV infections in West and Central Africa. The country also has one of the massive HIV epidemic globally (UNAIDS, 2020; WHO, 2020). Combination antiretroviral therapies (cART) have effectively suppressed viremia to undetectable levels, increasing survival and quality of life. It has also decreased infectiousness in infected individuals (Herout et al., 2016; Hanke, 2019).

However, just about half of infected individuals are on antiretroviral therapy. Sub-Sahara Africa has the lowest access to treatment despite being the highest hit region with the virus (UNAIDS, 2019). Access to treatment is unlikely to increase to optimal levels because of economic, social, and pharmacologic challenges associated with cART use (Hanke, 2019; Ndung’u et al., 2019). Moreover, cART is not curative as persons on treatment have to use drugs to control the emergence of latent reservoir HIV (immune escape and drug-resistant strains) for the rest of their lives (Papuchon et al., 2013; Hanke, 2019). Therefore, to eliminate the HIV epidemic, especially in African countries, there is the need to develop a safe, cost-effective, durable, and accessible HIV-1 vaccine(Ondondo et al., 2016; Ndung’u et al., 2019; Bekker et al., 2020; Lé tourneau et al., 2020).

Induction of broadly neutralizing antibodies is the mainstay of all protective anti-viral vaccines. However, this has been very difficult to generate for HIV-1 because of the virus genes’ plasticity (Ondondo et al., 2016; Hanke, 2019; Ndung’u et al., 2019). Studies have shown that broadly neutralizing antibodies develop very late in infection after the latent reservoir landscape is already established (Burton et al., 2004; Muema et al., 2017). Therefore, at present, two arms of universal HIV-1 vaccine research is ongoing, with a group focusing on generating robust neutralizing antibodies and the other on effective CTL immune responses. Findings from these fields will hopefully be merged into one universal HIV vaccine (Ondondo et al., 2016). Cytotoxic T Lymphocytes (CTL), commonly referred to as CD8 + T cells have been extensively shown to control HIV-1 replication, especially during the early stages of infection (Ndhlovu et al., 2015; Leitman et al., 2016; Ndung’u et al., 2019). Previous studies have associated expansion of CTL with the control of acute infections (Borrow et al., 1994; Koup et al., 1994; Ogg et al., 1998; Goulder and Watkins, 2004; Goonetilleke et al., 2009). Initially, several studies associated CTL control of acute HIV infections with HLA protection (Gillespie et al., 2002; Brumme et al., 2008; Honeyborne et al., 2010; Mori et al., 2014); however, recent studies have shown HIV/AIDS outcomes are better predicted by the (i) magnitude and breadth of CTL responses as well as (ii) presentation of functionally conserved CTL epitopes during the early stages of infection (Balla-Jhagjhoorsingh et al., 1999; Lazaro et al., 2011; Matthews et al., 2012; Kløverpris et al., 2014; Ndhlovu et al., 2015; Radebe et al., 2015; Koofhethile et al., 2016).

Recently, the conserved region T cell-based vaccine strategy was developed (Létourneau et al., 2007; Hanke, 2019). This strategy is aimed at eliciting effective CTL responses by (a) using functionally conserved HIV-1 proteins for vaccine constructs, (b) blocking common HIV immune escape paths, and (c) including epitopes associated with low viral load in untreated people (Ondondo et al., 2016; Hanke, 2019). Using proteomic and bioinformatics methods, which included the Shannon entropy algorithm, 14 highly conserved consensus HIV-1 proteins were assembled into a chimeric vaccine construct (Barouch et al., 2010; Santra et al., 2010; Borthwick et al., 2014; Abdul-Jawad et al., 2016). This construct induced high frequencies of HIV-1 specific T cells capable of inhibiting HIV-1 replication *in vitro* and rhesus monkeys (Barouch et al., 2010; Abdul-Jawad et al., 2016). The immune coverage of the construct against diverse HIV strains was also noted (Santra et al., 2010). The construct was further developed into just six functionally conserved regions, which spanned six areas, namely, the whole of Gag p24, one part in Gag p15, and four regions of Pol overlapping with protease, polymerase, and integrase (Ondondo et al., 2016). This second-generation vaccine construct was showed to possess strong immunogenicity in mice (Mothe et al., 2015). The construct also elicited CD8 T cells, which correlated with high CD4 T-cell count in untreated patients (Ondondo et al., 2016).

Three conserved epitopes out of the six, namely Gag 240–249, TSTLQEQIGT; Gag 162–172, KAFSPEVIPMF and Gag 203–212, ETINEEAAEW, were shown to be functionally conserved and possessed high immune coverage among diverse HIV-1 strains. One of these epitopes- Gag 162–172, KAFSPEVIPMF is found within the major homology region (MHR) of the HIV-1 Gag gene. CTL epitopes within the MHR are promiscuously presented by similar HLA alleles (Carlson et al., 2012). The MHR, found within the capsid gene, is a conserved motif among retroviruses (Honeyborne et al., 2010; Tanaka et al., 2016). This region is essential in particle assembly and viral infectivity (Reicin et al., 1995). Furthermore, CTL epitopes within the MHR are also hydrophobic, highly immunogenic, and immuno-dominant (Tenzer et al., 2009; Bennett et al., 2010; Kløverpris et al., 2015; Yang et al., 2015).

It is also hypothesized that escape mutations within the MHR are likely to be deleterious to HIV as they seem to be associated with fitness costs (Martinez-Picado et al., 2006; Troyer et al., 2009; Liu et al., 2014). Ondondo et al. and others identified Gag 240–249, TSTLQEQIGT, and Gag 203–212, ETINEEAAEW as functionally conserved epitopes with high coverage within HIV-1 diverse strains (Fischer et al., 2007; Abdul-Jawad et al., 2016; Ondondo et al., 2016). These epitopes also showed robust T cell immune responses when assembled in vaccine constructs and tested in macaques (Mothe et al., 2015; Ondondo et al., 2016). However, despite ongoing research in this field, insufficient data exists on these CTL epitopes’ functionally conserved abilities in early HIV-1 infected individuals’ in real-world scenarios. There is also a dearth of information on the rate of non-synonymous substitutions in these epitopes compared to other epitopes of the HIV-1 GAG region in natural settings. This knowledge gap is prominent in Sub-Saharan African countries where diverse HIV strains circulate.

These countries are also of very high priority in the development of HIV-1 vaccines (Ndung’u et al., 2019). When developed, HIV-1 vaccines must be effective against strains circulating in African countries (Ndung’u et al., 2019). Despite the West African epicenter having one of the highest numbers of diverse circulating HIV-1 strains, very few longitudinal studies on HIV-1 have been reported from the region. Escape due to CTL epitopes, which is driven by frequencies of non-synonymous and synonymous substitutions (Kosakovsky Pond et al., 2008; Garcia-knight et al., 2016), outside the functionally conserved region may be a crucial factor to consider in the design of therapeutic and universal HIV vaccines (Murakoshi et al., 2019). They may also provide opportunities for compensatory mutations on replicative fitness (Crawford et al., 2011).

This study describes non-synonymous substitutions within and without the three functionally conserved epitopes (HIV_gagconsv) of HIV-1 GAG genes isolated from 10 infected Nigerians at the early stage of HIV-1 infection. These individuals were part of a previously described study (Olusola et al., 2017). Furthermore, using phylogenetic tools, programs and databases in the Los Alamos National Laboratory HIV Sequence Database^1^, we analyze these substitutions longitudinally in five infected individuals from the early stages of infection until after seroconversion.

## Methods

### Study Sites and Patient Population

Twenty-three individuals at the early stages of HIV-1 infection identified and described previously were recruited for this study (see Table 1). Ten out of these individuals were studied for early HIV-1 infection. Another five individuals identified in 2017 were followed up until after seroconversion. The profile of the follow-up schedule is shown in Table 2. These individuals were screened for HIV-1 infection at every visit using the earlier described protocol (Olusola et al., 2017, 2020b).

**TABLE 1:** Summary of 23 early HIV infected Nigerians.

**Sample ID**	**Gender**	**Age(years)**	**High-Risk group**	**Location**	**Date of HIV detection**	**HIV-1 RNA Viral Load**	**Subtype**	**GenBank accesssion number**
EHIV001	Female	21	RM	Saki	26 Jan 2016	Undetectable	Unassigned	KY786266
EHIV002	Female	45	RM	Ibadan	26 Jan 2016	Undetectable	K	KY786267
EHIV003	Female	31	RM	Saki	26 Jan 2016	4999281	CRF 02-AG	KY786268
EHIV004	Female	32	RM	Saki	26 Jan 2016	5142127	CRF 02-AG	KY786269
EHIV005	Female	38	RM	Saki	26 Jan 2016	5856363	CRF 02-AG	KY786270
EHIV006	Female	28	RM	Saki	26 Jan 2016	1742364	Recombinant GD	KY786271
EHIV007	Male	48	RM	Ibadan	26 Jan 2016	Undetectable	CRF 02-AG	KY786272
EHIV008	Male	38	RM	Saki	26 Jan 2016	Undetectable		Not Sequenced
EHIV009	Female	32	RM	Ibadan	20 Feb 2017	Undetectable		Not Sequenced
EHIV010	Male	31	RM	Ibadan	21 Feb 2017	6492033	A	MN943617
EHIV011	Male	26	RM	Ibadan	21 Feb 2017	5449249	A	MN943616
EHIV012^*b*^	Female	29	RM	Ibadan	05 Oct 2017	5413537	G	MN943624
EHIV013^*b*^	Female	32	RM	Ibadan	21 Aug 2017	6906290	A	MN943613
EHIV014	Male	25	RM	Ibadan	21 Jul 2017	10084640	CRF 02-AG	MN943628
EHIV015	Male	42	VBD	Ibadan	26 Jul 2017	5499245	A	MN943615
EHIV016^*b*^	Male	29	VBD	Ibadan	02 Aug 2017	4013634	G	MN943627
EHIV017	Female	26	RM	Ibadan	11 Nov 2016	5613523	CRF 02-AG	MN943629
EHIV018	Female	29	RM	Ibadan	20 Feb 2017	5642093	CRF 02-AG	MN943633^*a*^
EHIV019	Male	46	VBD	Ibadan	27 Jul 2017	Undetectable		Not Sequenced
EHIV020	Male	40	VBD	Ibadan	17 Aug 2017	4392180	CRF 02-AG	MN943634^*a*^
EHIV021	Male	29	VBD	Ibadan	21 Jul 2019	Undetectable	CRF 02-AG	MN943635^*a*^
EHIV022^*b*^	Male	33	VBD	Ibadan	24 Jul 2017	6820582	G	MN943625
EHIV023^*b*^	Male	22	VBD	Ibadan	17 May 2017	^*c*^Undetectable	A	MN943619

**TABLE 2:** Analysis of samples collected during follow- up.

			**Follow-up Schedule**					
**S/N**	**Sample ID**			**Early HIV infection stage**				**Detection of Abs^#^**
		**Accession Numbers**	**Baseline**	**3months**	**6months**	**9months**	**1 year**	**∼2 years**
1	EHIV 012	MN943626; MN943624; MN943630	05/10/2017^a^		✓			✓
2	EHIV 013	MN943613; MN943614; MN943631	21/08/2017^a^		✓		✓	✓
3	EHIV 016	MN943627; MN943623	02/08/2017^a^		✓			✓
4	EHIV 022	MN943625; MN943622	24/07/2017^a^		✓			✓
5	EHIV 023	MN943618; MN943619; MN943620; MN943621	17/05/2017^a^			✓	✓	✓

***^a^** HIV-1 GAG gene isolated and sequence.*

*^#^ Antibodies detection does not correspond to seroconversion dates as these individuals had seroconverted at an earlier date.*

### Recruitment of Participants, Sample Collection, and Processing

Participants were recruited for this study after obtaining informed consent. Experiments were conducted with the understanding and the consent of participants. Socio-demographic data of participants were collected using a structured questionnaire. Feedback on results was provided within a week of sample collection. Individuals were counseled and encouraged to continue the presentation for testing at the scheduled intervals. Five milliliters of whole blood were collected in EDTA bottles from participants at every visit. Plasma was separated from the samples immediately after collection, stored at −20°C, and transported in a cold chain to a central laboratory for analysis. The samples were then stored at −80°C until analyzed. Blood samples were analyzed for HIV antigen/antibody, serum creatinine, HIV-1 RNA viral load (at baseline), and HIV-1 GAG DNA.

### Identification of Early HIV-1 Infection and Detection of Antibodies

The updated CDC algorithm of laboratory testing for the diagnosis of early and chronic HIV-1 infection was used for this study, as previously described (Olusola et al., 2017).

### Clinical Chemistry Assay for Serum Creatinine

Plasma samples were analyzed on a Roche cobas^®^ C11 blood chemistry analyzer (Roche Diagnostics, Indianapolis, United States). Each sample was analyzed to determine the level of serum creatinine, according to the manufacturer’s instruction. Normal reference ranges for plasma creatinine is 62–133 μmol/L.

### HIV-1 RNA Viral Load Testing

Serum samples collected at baseline were tested for Plasma HIV-1 viral load (copies/ml) using the COBAS^®^ Ampliprep/COBAS TaqMan96^®^HIV-1 Test, v2:0 (Roche Molecular Diagnostics, Branchburg, NJ, United States) according to manufacturer’s instruction or by an in house real-time PCR protocol. Briefly, the in-house real-time PCR protocol entails a two-step reaction. First, reverse transcription PCR for cDNA which is a 25 μL reaction utilizing 5 μL of extracted RNA, 12.5 μL of 2X superscript III-RT polymerase reaction mix, and 1 μL superscript III RT/Platinum Taq high fidelity mix (Jena Bioscience, Jena, Germany) as well as random hexamers (1 μL) and RNase free water (5.5 μL). Thermal cycling was performed at 20°C for 10 min, 45°C for 30 min, 70°C for 15 min and an RNase H step of 37°C for 20 min using Applied Biosystem 7500 Fast Real-Time PCR system (Thermo Fisher Scientific, MA, United States). The second stage involved a quantitative real-time PCR targeting a 140bp *Nef- Env* region of HIV-1. The 20 μL reaction utilized 5 μL of cDNA, 10 μL of qPCR SYBR Master UNG (Jena Bioscience, Jena, Germany) as well as 0.6 μL each of Nef8343 (ATGGGTGGCAAGTGGTCAAAAG) (Tcherepanova et al., 2008) and Env3out (TTGCTACTTGTGATTGCTCCATGT) primers (Keele et al., 2008). Inqaba Biotechnology, South Africa synthesized the primers, and thermal cycling was performed at 50°C for 2 min, 95°C for 2 min and then 35cycles of 95°C for 15 s, 55°C for 20 s using Applied Biosystem 7500 Fast Real-Time PCR system (Thermo Fisher Scientific, MA, United States). Quantitation standards were used to interpolate the quantitative values of the HIV-1 RNA viral load for samples.

### PCR Amplification and Sequencing of the GAG Gene

Total DNA was extracted from whole blood samples collected at each visit using guanidium thiocyanate in house protocol. A fragment of the gag-pol region (900 base pairs) of the virus was amplified using previously published primers and cycling conditions by Gall 2012 (Gall et al., 2012) with slight modifications. Briefly, PCR was performed using platinum TaqDNA High fidelity polymerase (Jena Bioscience). Each 25 μl reaction mixture contained 12.5 μl reaction mix (2x), 4.5 μl RNase-free water, 1 μl each of each primer (20 pmol/μl), 1 μl Platinum Taq DNA High Fidelity mix, and 5 μl of template DNA. Pan-HIV-1_1R (CCT CCA ATT CCY CCT ATC ATT TT) and Pan-HIV-1_2F (GGG AAG TGA YAT AGC WGG AAC) were used. Cycling conditions were 94°C for 5 min; 35 cycles of 94°C for 15 s, 58°C for 30 s, and 68°C for 1 min 30 s; and finally, 68°C for 10 min. Positive HIV samples that were undetectable using the above-stated primers were retested using another set of GAG primers for nested PCR as described previously (Vidal et al., 2000). Positive PCR reactions were shipped on ice to Macrogen, South Korea, for Big Dye sequencing using the same amplification primers (Pan-HIV-1_1R and Pan-HIV-1_2F; or G60 andG25).

### Detection of HIV-1 Subtypes and Phylogenetic Analysis

The sequences were cleaned and edited using Chromas and Bioedit software. Subtyping was performed using a combination of four subtyping tools: The Rega HIV-1 Subtyping Tool, version 3.0^2^, Comet, version 2.2^3^, National Center for Biotechnology Information, Bethesda, MD^4^ and jpHMMM: Improving the reliability of recombination prediction in HIV-1^5^. The first three tools were used simultaneously, while jpHMMM was used to resolve discordant subtypes. Phylogenetic analyses were performed using MEGA software version 10. Alignment of sequences was performed using MAFFTS online software. Genetic distances were inferred using the Tamura-Nei model, and a phylogenetic tree was generated using the maximum likelihood method. The robustness of the tree was evaluated with 1000 bootstrap replicates. All consensus nucleotide sequences obtained in this study were submitted to GenBank database and assigned accession numbers MN943617-635.

### Non-synonymous Substitutions in Cytotoxic T Lymphocytes (CTL) Epitopes Within HIV_gagconsv of HIV-1 GAG Gene Isolated From 10 Early Infected Individuals

Reference GAG sequences for subtypes G, A, and CRF02-AG were downloaded from the Los Alamos National Laboratory HIV Sequence Database^6^. Deduced amino acid (aa) sequences were translated for both reference and sample sequences with the standard genetic code using Bioedit software. CTL epitope corresponding to the three highly conserved sites (HIV_gagconsv), namely KAFSPEVIPMFSALSEGATPQD, DTINEEAAEWDR, and TSTLQEQIR (Yang et al., 2015; Ondondo et al., 2016; Hanke, 2019; Lé tourneau et al., 2020), were used for comparison and identification of amino acid substitution. HIV-1 GAG sequences identified as subtypes A, G, and CRF02_AG in this study were aligned with Reference A(GenBank accession numbers DQ676872; AB253421 and AB253429), G(GenBank accession numbers AF084936; AF061641; U88826 and AY612637), and CRF02_AG(GenBank accession numbers L39106 and DQ168578) sequences respectively.

### Non-synonymous Substitutions in Cytotoxic T Lymphocytes (CTL) Epitopes Outside the HIV_gagconsv of HIV-1 GAG Gene

The Virus Epidemiology Signature Patterns Analysis^7^ program was used to identify variations in other sites HIV-1 GAG sequence outside the **HIV_gagconsv** corresponding to CTL epitope regions (Korber and Myers, 1992). Already defined CTL epitope in HIV-1 database can be found in https://www.hiv.lanl.gov/content/immunology/ctl_search. The VESPA program is a user-friendly amino and nucleic acids signature pattern analysis tool. The program can calculate numbers of variations in an amino acid sequence relative to background sequence(s) using bioinformatics algorithms. By selecting positions where the most common character in a query set differs from that in the background set, differences between groups of sequences can quickly be detected. This analysis invariably can also identify conserved sequence signature patterns. The frequencies of distinguishing amino acids in each set can also be determined. The program has previously been used for HIV-1 and Chikungunya sequence analysis (Ou et al., 1992; Salvatierra and Florez, 2017). This program can also distinguish non-synonymous substitutions from synonymous substitutions based on the threshold settings. Similarity scores or thresholds represent Hamming distances (Nowak et al., 1991) or the number of point mutations between two aligned sequences, calculated using the score: (1 — D) X 100% where D is the hamming distance. This algorithm has previously been described in Nowak et al. (1991), Korber and Myers (1992).

With a threshold setting of 100%, only non-synonymous substitutions that are not due to chance were reported in this study. Sequences were aligned using CLC Main Workbench version 6.5, after which VESPA analysis was performed.

Deduced amino acid (aa) sequences were translated for both reference and sample sequences with the standard genetic code using Bioedit. HIV-1 GAG sequences identified as subtypes A, G, and CRF02_AG in this study were aligned with Reference A (GenBank accession numbers DQ676872; AB253421 and AB253429), G (GenBank accession numbers AF084936; AF061641; U88826 and AY612637), and CRF02_AG (GenBank accession numbers L39106 and DQ168578) sequences respectively. Only amino acid replacements with 100% non-synonymous substitution between reference sequences and sample sequences were considered. Percentage substitution rates were calculated by finding the ratio of the number of substitutions to the total possible substitution sites. These mutations were compared with the Los Alamos National Laboratory HIV Immunology Database for CTL/CD8 + Epitope Variants and Escape Mutants^8^.

### Ethical Approval

This research was conducted following the declaration of Helsinki. Experiments were conducted with the understanding and the consent of each participant. Ethical approvals for this research were obtained from the University of Ibadan/University College Hospital (UI/UCH) Research and Ethics Committee (UI/EC/15/0076) and the Oyo State Ministry of Health Committee on Human Research (AD13/479/951). All results were delinked from patient identifiers and anonymized.

### Eligibility/Exclusion Criteria

Only individuals between 18 and 65 years of age were included in the study. Individuals who already knew their HIV status were excluded from the study.

### Data Management and Statistical Analysis

Statistical analyses were performed using SPSS version 20. Data are expressed as means ± standard deviations. Statistical significance was estimated using the Kruskal-Wallis test, with SPSS package version 12.0, while Statistical significance was defined as *P*-values = 0.05.

## Results

### Participants’ Characteristics

**FIGURE 1:**
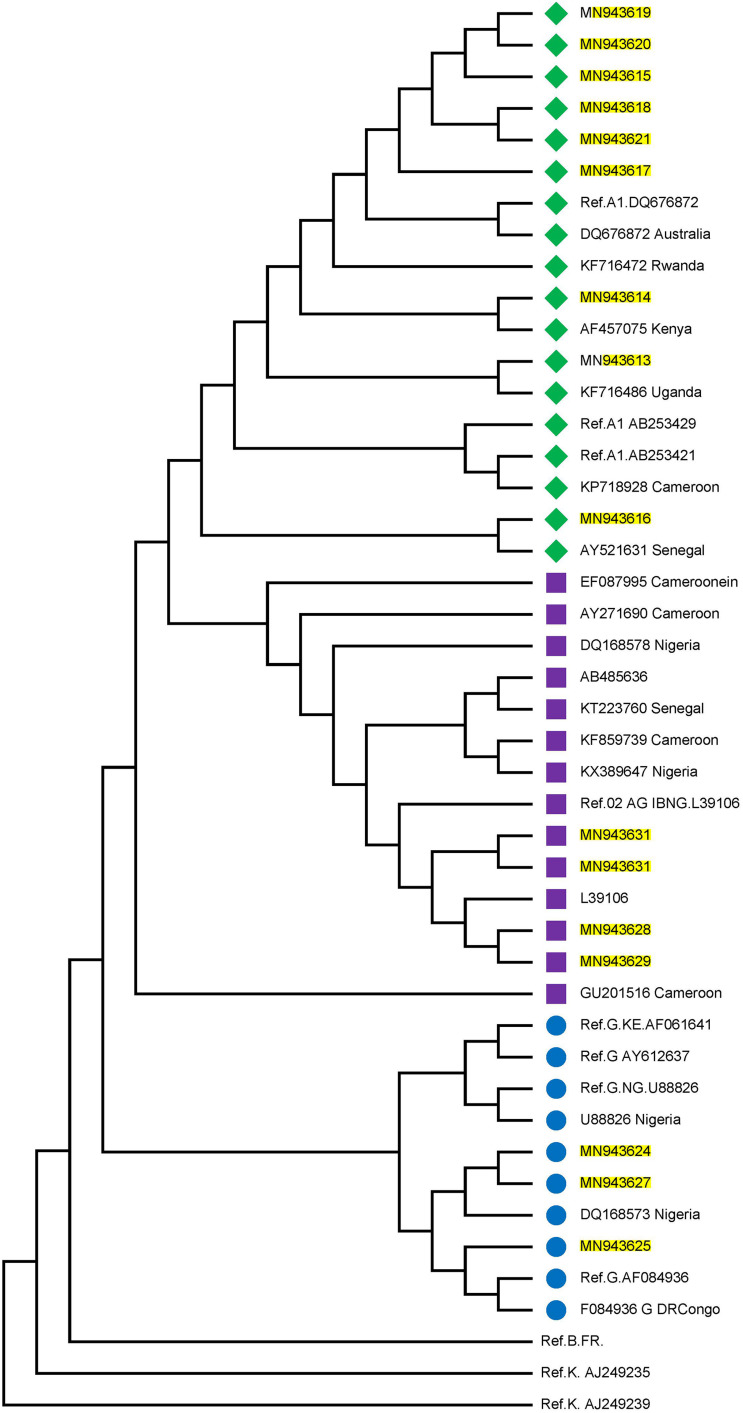
Phylogenetic tree of the P17/P24 regions of the GAG gene of HIV-1. Reference subtypes are indicated with Ref. before their accession numbers. Other sequences are shown with their accession numbers and country of isolation. Subtypes obtained from samples in this study are shown with their accession numbers only. Subtypes A, G, and CRF02_AG were identified with green diamond, blue circle, and pink square symbols respectively. Multiple sequence alignment and phylogenetic tree were constructed using MAFFTS and Maximum Parsimony algorithm in MEGA 6 software. Statistical significance of the tree topology was tested by 1000 bootstrap replication.

Twenty-three individuals were identified to be at the early stages of HIV-1 infection. Figure 1 shows the phylogeny of HIV-1 subtypes. Out of the ten early infected persons studied, five were infected with Subtype A, three with subtype G, and the rest CRF02-AG. Five individuals at the early stages of HIV-1 infection were followed up until after seroconversion. However, samples in which antibodies were detected were collected after these individuals had seroconverted. The participants were identified to be at the early stages of infection at different periods in 2017. One individual in October, July, and May each and two in August. Three of the individuals were males and were voluntary blood donors. The remaining two females were identified when referred for malaria antigen test. Three individuals were infected with HIV-1 subtype G, while the other two were infected with subtype A (Table 1).

Table 2 shows the analysis of the samples collected from these individuals from baseline until after seroconversion. In four individuals, blood samples were collected at three time points, twice during the early stages of infection and once after seroconversion. Samples were collected four times in the fifth individual, thrice during the early stages of infection and once after seroconversion. HIV-1 GAG sequences of these infected individuals were determined at every time point of blood collection.

### Phylogenetic Analysis

Figure 1 shows the estimated phylogeny of HIV-1 subtypes with respect to reference sequences available in the HIV Los Alamos National HIV Sequence database. As shown in the Figure, Subtypes A, G, and CRF02_AG were identified with green, blue, and pink symbols, respectively. HIV-1 subtypes A identified in this study were closely related to Ref A1 DQ676872 (from Nigeria) and subtypes AF457075, KF716486 and AY521631 from Kenya, Uganda and Senegal, respectively. Those identified as subtypes G and CRF02-AG were closely related to the Nigerian subtypes DQ168573 and Ref.02 AG IBNG. L39106 respectively.

### Substitutions in HIV_gagconsv of CTL Epitope Regions of Subtype A HIV-1 GAG Gene During Early Infection

We compared intra and inter variations among 10 HIV-1 GAG sequences isolated from persons at the early stage of infection. These sequences were grouped by subtypes and analyzed alongside reference sequences. As shown in Table 3, variations occurred mostly in HIV-1 Subtype A at the CTL epitope region of 243-251aa. The conserved epitope of TSTLQEQIR was not found in both the reference subtypes and those from early infected individuals. HIV-1 Subtype A also had the highest variations (50%) for HIV_gagconsv corresponding to the CTL epitope region (203-214aa). Although the HIV_gagconsv for CTL epitope region 162-183aa was the most conserved among the subtypes, a substitution rate of 2.7% was found for HIV-1 subtype A isolates. The CTL escape region (162-183) KAFSPEVIPMFSALSEGATPQD had the lowest frequency of mutations. However, sample MN943615 had two mutations-K162R and A163G, while MN943616 had mutation A163G only.

**TABLE 3:** Substitutions associated with HIV_gagconsv in HIV-1 GAG gene during early HIV infection.

	**EHIV accession no**	**CTL epitope region** (162-183)	**CTL epitope region (203-214aa)**	**CTL epitope region (243-251aa)**	**P-value**
	Ref Seq	KAFSPEVIPMFSALSEGATPQD	DTINEEAAEWDR	TSTLQEQIR	
**G**	MN943624	Conserved	Conserved	Conserved	ns
	MN943625	Conserved	D203E	Conserved	
	MN943627	Conserved	Conserved	Conserved	
	% Substitution	Nil	2.7	Nil	
	Ref Seq			*TSTPQEQLQWMT	
**A**	MN943613	Conserved	Conserved	TSTTQEQIAWMT	
	MN943615	K162R; A163G	DTSPTR*PWAGI	*HXQELLVPLK	ns
	MN943616	A163G	DTSMRKLQNGTD	*HXQELLVPLK	
	MN943617	Conserved	Conserved	*HXQELLVPLK	
	MN943619	Conserved	DTIN*G*PXXGQ	*HNRSY*YPSRT	
	% Substitution	2.7	50	65	
**CRF-02 AG**	MN943628	Conserved	Conserved	Conserved	ns
	MN943629	Conserved	Conserved	Conserved	
	% Substitution	Nil	Nil	Nil	
***P*-Value**		ns	ns	**0.00902**	

*P = 0.00902 for comparisons of non-synonymous substitutions in sequences across the CTL epitope TSTLQEQIR group.*

### Sequences Isolated After Seroconversion Are Associated With a Higher Rate of Substitutions in HIV_gagconsv of CTL Epitope Regions

Substitutions in the HIV_gagconsv of subtype B HIV-1 GAG gene have been shown to come with a fitness cost. These substitutions have shown to be deleterious to HIV-1 strains’ eventual survival and transmission. However, minimal information exists on substitutions associated with immune epitopes during early non-subtype B HIV-1 infection. We followed five individuals from the early stage of infection till after seroconversion. Three of these individuals were infected with subtype G, while the remaining two were subtypes A (see Table 4). There were no substitutions in CTL epitope regions for EHIV016, while EHIV022 had single aa substitutions before and after seroconversion.

**TABLE 4:** Substitutions associated with HIV_gagconsv in HIV-1 GAG gene isolated from 5 individuals followed from early HIV infection till after seroconversion.

**Subtype**	**Sample ID**	**EHIV accession no**	**CTL epitope region** (162-183)	**CTL epitope region (203-214aa)**	**CTL epitope region (243-251aa)**	
		**Ref Seq**	**KAFSPEVIPMFSALSEGATPQD**	**DTINEEAAEWDR**	**TSTLQEQIR**	***P*-Value**
G	EHIV016	MN943627	Conserved	Conserved	Conserved	ns
		MN943623^*a*^	Conserved	Conserved	Conserved	
		% Substitution	Nil	Nil	Nil	
	EHIV022	MN943625	Conserved	D203E	Conserved	ns
		MN943622^*a*^	Conserved	E203D	Conserved	
		% Substitution	Nil	4	Nil	
	EHIV012	MN943626	Conserved	Conserved	Conserved	ns
[1pt]		MN943624	Conserved	Conserved	Conserved	
		MN943630^*a*^	M175k	D207E	R251G	
		% Substitution	2	2	2	
		Ref Seq			TSTPQEQLQWMT	**0.04929**
A	EHIV023	MN943619	Conserved	DTIN*G*PXXGQ	*HNRSY*YPSRT	
		MN943618	Conserved	Conserved	*HSRSY*YPSRT	
		MN943620	Conserved	DTINEEEFG*SN	*HSRSY*YPSRT	
		MN943621^*a*^	Conserved	Conserved	*HSRSY*YPSRT	
		% Substitution	Nil	31.2	8.3	
	EHIV013	MN943613	Conserved	Conserved	TSTTQEQIAWMT	ns
		MN943614	Conserved	Conserved	TSTPQEQIGWMT	
		MN943631^*a*^	A166G	L216V	TSTLQEQIGWMT	
		% Substitution	2.7	2.7	16.6	
	*P*-Value		ns	ns	ns	
		^*a*^Sequence isolated after seroconversion				

*There were significant differences (P = 0.04929) in non-synonymous substitutions within EHIV023 sequences for the three functionally conserved epitopes.*

EHIV012 had single aa substitutions each after seroconversion in the three CTL epitope regions studied. These substitutions have not been reported before, to the best of our knowledge. For HIV-1 subtype A samples, EHIV023 had very high substitution rates in the CTL epitope region of 203-214aa (31.2%), although there were reversions after seroconversion. Amino acid substitutions were also observed in the CTL epitope region spanning 243-251aa (8.3%). Significant differences (*P* = 0.04929) in substitution rates before and after seroconversion across two of the three CTL epitope regions were observed for the EHIV023 sample (see Table 4). Substitutions were more associated with the HIV-1 GAG gene sequenced after seroconversion for EHIV013 across the three CTL epitope regions studied. The two amino acid substitutions, A166G and L216V, observed in EHIV013, occurred after seroconversion and were not previously reported.

### Amino Acid Signature Patterns in Variable Sites of HIV-1 GAG Genes

In this study, only sequences with non-synonymous substitutions compared to the reference sequence were analyzed. In Table 5, these substitutions were compared between the reference sequence and sequences isolated from individuals in the early stages of infection. In contrast, in Tables 6, 7, the substitutions were compared within sequences isolated per sample spanning early HIV infection till after seroconversion. As shown in Table 5, non-synonymous substitutions were mostly observed in subtype G. However, a substitution, E105K, observed in CRF02_AG had been previously identified as a variant not recognized by the HXB2 epitope (Li et al., 2007). Subtype A had a frameshift mutation at aa85-88. There were no substitutions in sequences isolated from sample EHIV016, while EHIV 012 had the highest substitution within Subtype G sequences (see Table 6). EHIV022 and EHIV 012 had substitutions within sequences at aa positions of 106 and 110-113.

**TABLE 5:** Frequency of non-synonymous substitutions in sites outside the HIV_gagconsv of HIV-1 GAG gene amino acids.

	**aa position in HIV-1 GAG gene**	**16**	**21**	**34**	**68**	**70**	**103**	**107**	**%sub**
G	aa in Ref seq	L	V	Y	S	X	G	D	
	aa in EHIV samples	X	M	N	G	Y	C	V	2.3
	aa position in HIV-1 GAG gene	92	97	105	116	176			
CRF02 AG	aa in Ref seq	L	T	E	Q	T			
	aa in EHIV samples	Q	K	K	I	K			1.6
	aa position In HIV-1 GAG gene	89							
A*	aa in Ref seq								
	aa in EHIV samples	X							

These aa replacements were 100% substituted in the EHIV samples compared to Ref seq. *Indel in aa positions 85-88 introduced a frameshift.

**TABLE 6:** Frequency of non-synonymous substitutions in variable sites outside the HIV_gagconsv of HIV-1 Subtype G GAG gene isolated from 3 individuals followed up from early infection till after seroconversion.

**Sample ID**	**Accession no**												
EHIV016	MN943627		No variable site										
	MN943623^*a*^												
EHIV022 (2.3% sub)	aa position	106	107	108	110-113								
	MN943625	N	V	C	RREK								
	MN943622^*a*^	K	I	Q	KSQE								
EHIV012 (8.6%sub)	aa position	31	62	66	69	82	86	90-96	101	104	106	110-114	121-2
	MN943626	M	T	L	Q	V	Y	QEFWLKG	V	V	K	KSQQE	GN
	MN943624	M	K	P	Q	V	Y	QRMGVKD	V	V	K	KSQQE	GN
	MN943630^*a*^	L	E	S	R	I	W	QRIDIRD	L	M	E	KSKQK	GN
	aa position	158	170	172	185	189	206	210	214	217	224	248	
	MN943626	V	M	T	T	T	D	E	L	TQ	A	R	
	MN943624	V	M	T	T	I	D	E	T	TQ	A	R	
	MN943630^*a*^	I	K	S	X	I	E	D	T	VH	P	G	

*These aa replacements were 100% substituted in the early HIV-1 infected samples compared to ^*a*^sequences from the same persons isolated after seroconversion.*

**TABLE 7:** Frequency of non-synonymous substitutions in variable sites outside HIV_gagconsv of HIV-1 Subtype A GAG gene isolated from 2 individuals followed up from early infection till after seroconversion.

**Sample ID**	**Accession no**													
**EHIV023** (4% sub)	**aa position**	**205**	**215-222**	**228-230**										
	MN943619	I	LHPVHAGX	DKR										
	MN943618	I	QG*LYPRC	PAX										
	MN943620	T	NG*LYPRC	DKX										
	MN943621^*a*^	I	LHPVHAGX	DKR										
**EHIV013** (13.6%sub)	**aa position**	**15**	**28**	**30**	**41**	**49**	**60**	**62-62**	**66**	**69**	**72**	**76**	**82**	**86**
	MN943613	K	T	K	M	G	V	EQ	S	K	T	R	V	Y
	MN943614	S	K	R	L	S	I	DR	P	K	S	R	V	Y
	MN943631^*a*^	A	K	R	L	G	L	EQ	S	R	S	K	I	W
	**aa position**	**92-93**	**104**	**107**	**115**	**118**	**121**	**124**	**125-127**	**139**	**143**	**146-7**	**182**	**215**
	MN943613	EV	I	I	E	A	D	N	SKV	Q	V	AV	G	L
	MN943614	DV	I	M	P	A	D	S	SKV	Q	V	TL	Q	L
	MN943631^*a*^	DI	M	I	V	T	A	S	***	R	T	SM	Q	V
	**aa position**	**218**	**223-224**	**228**	**243**	**248**	**252**	**260**	**263**	**267**				
	MN943613	L	IA	I	T	A	G	D	R	I				
	MN943614	V	VP	M	P	G	S	D	K	I				
	MN943631^*a*^	V	IP	M	L	G	S	E	K	V				

*These aa replacements were 100% substituted in the EHIV samples compared to ^*a*^Sequence isolated after seroconversion. The red letters indicate sequence reversion after seroconversion.*

Lysine was the commonest aa used at position 106 (3/5), while a single substitution was observed for glutamate and asparagine. KSQ was the commonest aa usage in positions 110-113 (3/5); other aa in these positions were RRE (1/5) and KSK (1/5). In Table 7, non-synonymous substitutions associated with HIV-1 subtype A are described. EHIV013 had the highest number of substitutions spanning aa region 15 to 267 in this study. Samples EHIV023 and EHIV013 had substitutions within sequences at aa positions 215, 218, and 228. In position 215, Leucine was the highest aa used (4/7), while glutamine, asparagine, and valine were present in one sequence each. Valine (4/7) and Leucine (3/7) were the only aa used in position 218. Aspartate (3/7) was the highest aa used in position 228, while methionine (2/7), proline (1/7), and isoleucine (1/7) were also present in some isolates.

### Non-synonymous Substitutions Associated With Immune Escape Variants Are More Within Epitopes Outside the HIV_gagconsv

**FIGURE 2:**
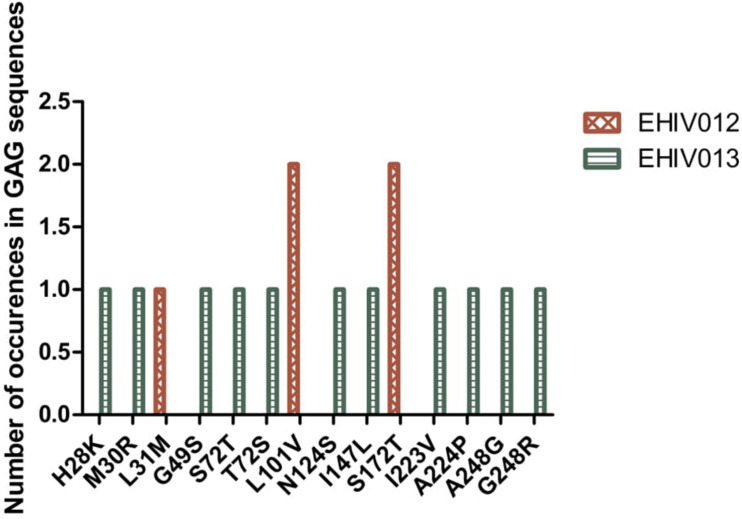
Distribution of mutations associated with escape in EHIV012 and EHIV013 before seroconversion. Three individuals had mutations previously associated with escape, diminished responses, non-susceptible forms, etc. These mutations were compiled from the Los Alamos National Laboratory HIV Immunology Database for CTL/CD8 + Epitope Variants and Escape Mutations^8^. The list of all the identified mutations is presented in Supplementary Table 1. Two individuals (EHIV012 and EHIV013) had mutations outside the GAG HIV_GAGCONSV, while EHIV022 had a mutation corresponding to escape (Murakoshi) – E203D. Figure 3 shows the distribution of mutations associated with escape in EHIV 012(Red Bars) and EHIV013 (Green Bars). The number of occurrences of the mutations in GAG sequences is shown in the Y-axis while the X-axis shows aa mutations.

**FIGURE 3:**
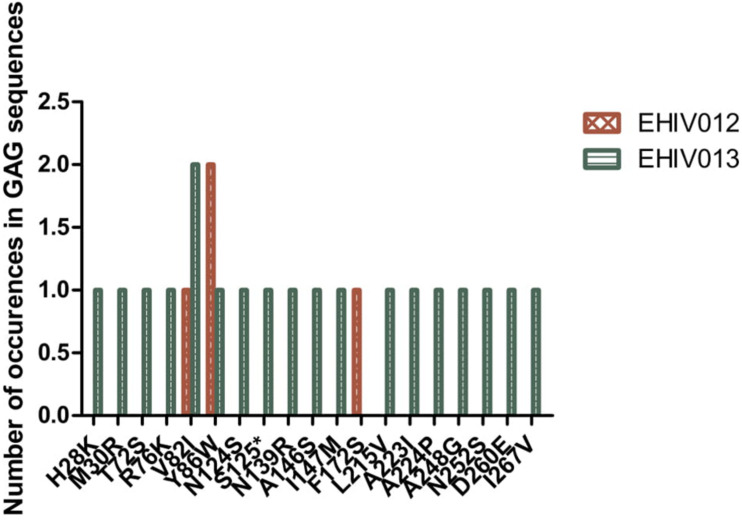
Distribution of mutations associated with escape in EHIV012 and EHIV013 after seroconversion. Three individuals had mutations previously associated with escape, diminished responses, non-susceptible forms, etc. These mutations were compiled from the Los Alamos National Laboratory HIV Immunology Database for CTL/CD8 + Epitope Variants and Escape Mutations^8^. The list of all the identified mutations is presented in Supplementary Table 1. Two individuals (EHIV012 and EHIV013) had mutations outside the GAG HIV_GAGCONSV, while EHIV022 had a mutation corresponding to escape (Murakoshi) – E203D. Figure 4 shows the distribution of mutations associated with escape in EHIV 012(Red Bars) and EHIV013 (Green Bars). The number of occurrences of the mutations in GAG sequences is shown in the Y-axis while the X-axis shows aa mutations.

Although four substitutions (E105K/CRF02AG; E203D/subtype G; K162R and A163G/subtype A) previously associated with immune escape were observed in HIV_gagconsv, more substitutions were found in regions outside the HIV_gagconsv. Out of the five individuals followed up, two had substitutions previously associated with immune escape strains in GAG gene sites outside the HIV_gagconsv (see Figures 2, 3). Most mutations associated with previously described immune escape strains were identified after seroconversion in this study. Fourteen mutations in 16 HIV-1 GAG sites outside the HIV_gagconsv were identified before seroconversion, while 21 mutations in 23 HIV-1 GAG sites outside the HIV_gagconsv were identified after seroconversion. As shown in Figures 2, 3, EHIV012 had three mutations before (L31M, L101V, and S172T) and after (V82I, Y86W, and F172S) seroconversion. H28K, M30R, A224P, and A248G mutations were identified pre and post seroconversion, while V82I and Y86W mutations were identified only after seroconversion.

### Serum Creatinine Concentration During Longitudinal Follow Up

**FIGURE 4:**
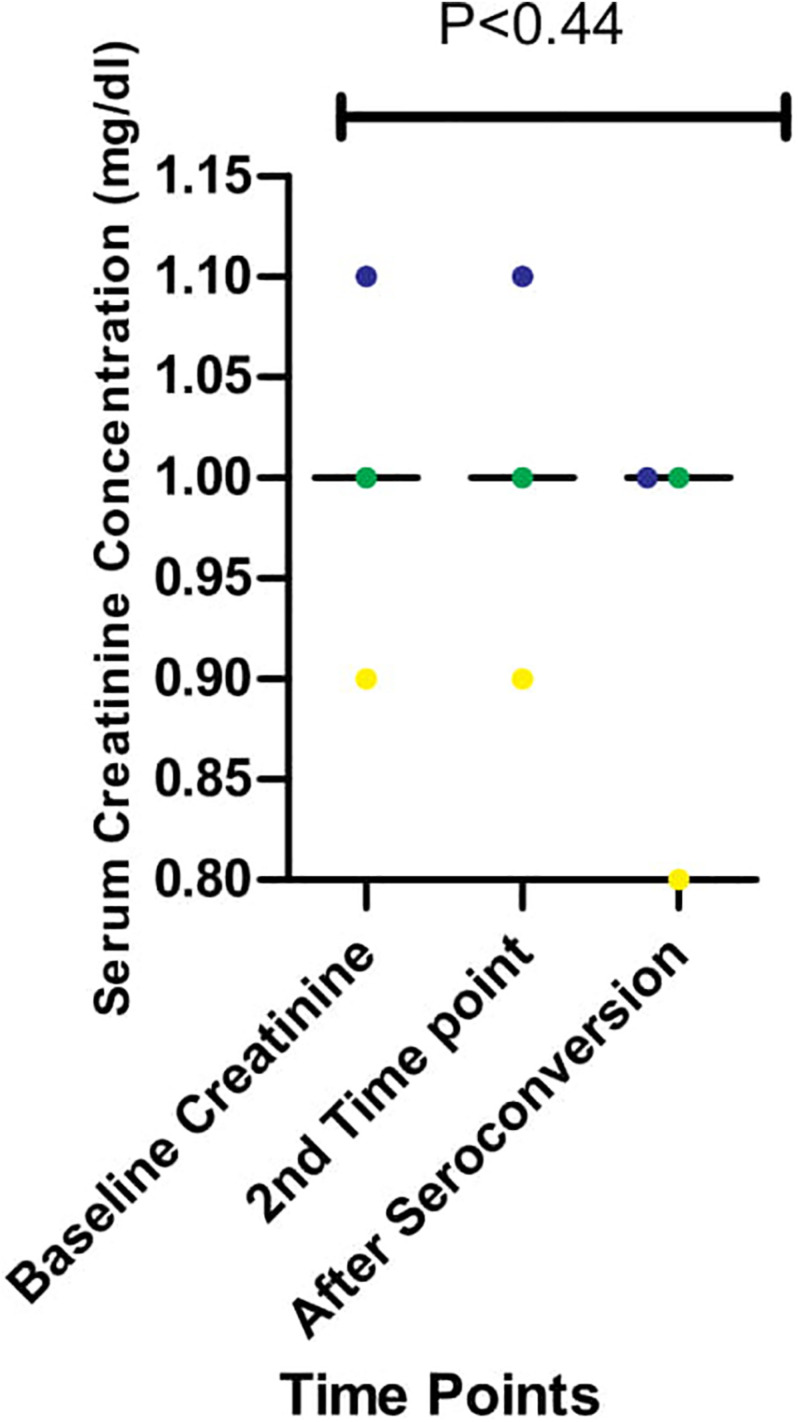
Serum creatinine concentration during longitudinal follow up of three individuals from early infection till after seroconversion. The three individuals followed up are represented in blue, yellow, and green. Serum creatinine levels for these individuals were measured in three-time, Baseline, second-time, and after seroconversion. Significant differences in the levels of serum creatinine (measured in mg/dl) observed across the three-time points (*P* < 0.44) were calculated using 1-way ANOVA.

We had earlier reported high levels of creatinine among HIV-infected Africans and African-Americans during the early stages of infection (Olusola et al., 2017, 2020b). In one of these studies (Olusola et al., 2020b), we also showed that immune activation of CTL was ongoing and correlated positively with high creatinine levels in early HIV-1 infected individuals. However, the effect of antibodies on creatinine levels were not observed in the previous study (Olusola et al., 2020b). As shown in Figure 4, there were differences in serum creatinine concentrations between early HIV-1 infection and seroconversion for the three individuals studied among the five persons followed up. EHIV 023 had lowest serum creatinine concentration at baseline (0.9 mg/dl) and after seroconversion (0.8 mg/dl) while EHIV 022 had the highest serum concentration of creatinine at baseline (1.1 mg/dl) and after seroconversion (1.0 mg/dl). Sample EHIV016 had no serum creatinine concentration changes from baseline until after seroconversion (1.0 mg/dl).

## Discussion

This study shows that diverse HIV-1 subtypes circulate in Nigeria, as subtypes A, G, and CRF02_AG were identified. Our study identified three non-synonymous substitutions within the HIV_gagconsv of HIV-1 GAG genes isolated from 10 early infected Nigerians. One substitution was, however, observed outside the HIV_gagconsv epitopes. Three (E105K, K162R, and A163G) of these substitutions have been previously related to immune escape (Currier et al., 2005; Li et al., 2007). These substitutions were associated with subtypes A and CRF02_AG. Most subtype G substitutions within the HIV_gagconsv were related to periods after seroconversion, while subtype A with early HIV infection.

Although few, substitutions within the HIV_gagconsv is a significant call for concern. Recent T cell-based vaccine studies have reported the likelihood of a single substitution within HIV_gagconsv affecting the immunogenicity vaccine constructs (Ondondo et al., 2016; Hanke, 2019). Immunogenicity of functionally conserved epitopes is a foundational requirement for an effective universal T cell-based HIV-1 vaccine (Létourneau et al., 2007). However, studies have shown that D203E mutations in the ETINEEAAEW epitope do not impact the epitope’s function and strain diversity coverage (Ondondo et al., 2016). Mutations after seroconversion may be due to the pressure of viral evasion mechanisms such as NEF mediated evasion of antibodies and HIV-1 subtype differences (Buckheit et al., 2012; Omondi et al., 2019). Studies have shown that these factors may account for replication and increased viremia during HIV-1 infection (Buckheit et al., 2012; Omondi et al., 2019).

We observed fourteen and nineteen mutations previously associated with immune escape outside the HIV_gagconsv before and after seroconversion, respectively, in this study. This finding is a far cry from the four observed within the HIV_gagconsv. It is also in accordance with previous reports that observed minimal non-synonymous substitutions in CTL epitopes within the HIV_gagconsv (Amicosante et al., 2002; Lazaro et al., 2011; Ondondo et al., 2016). APOBEC-3G has previously been noted as a major cause of hypermutation in the HIV-1 proviral landscape during the early stages of infection (Lee et al., 2019). Identifying these functionally conserved epitopes in our study participants lends credence to their immunodominance and high strain diversity coverage (Ondondo et al., 2016; Shu et al., 2020). However, the high rate of non-synonymous substitutions outside the HIV_gagconsv epitopes implies that escape mutants outside the HIV_gagconsv are more likely to be integrated into the HIV-1 latent cellular reservoir landscape. This is because latent reservoir strains are established during the early stages of infection and are known to compose majorly of CTL immune escape strains (Deng et al., 2014; Gounder et al., 2015; Roberts et al., 2015; Leitman et al., 2017). Therefore, these latent reservoirs may require a broad CTL response for clearance, as previously alluded to Deng et al. (2014). If this is the case, then using functionally conserved HIV-1 DNA vaccines for therapeutic clearance of latent reservoirs (hybrid HIV-1 cure) may be very difficult.

Therapeutic HIV-1 vaccines are intended to be used after cART stoppage, particularly against latent reservoir strains. However, as shown in this study, immune escape strains generated during the early stages of infection, which are likely significant constituents of the latent reservoir, may lead to therapeutic vaccine failures (Daucher et al., 2008; Ondondo et al., 2016; Hanke, 2019; Lé tourneau et al., 2020). Since immune escape occurs during the early stages of infection, cART must commence early to reduce the reservoir size and the incorporation of immune escape variants into the reservoir landscape (Brockman et al., 2015; Takata et al., 2017). Other studies have alluded that post-treatment control may be possible if treatment commences at the early stages of HIV infection since blips observed after cART stoppage are mainly due to immune escape variants incorporated into reservoir cells (Conway and Perelson, 2014). However, rapid and high magnitude CTL responses observed during the early stages of infection (Ndhlovu et al., 2015) may be affected by early treatment (Takata et al., 2017; Lee et al., 2019; Ndhlovu et al., 2019). This treatment can impair subsequent CTL responses during cART stoppage in post-treatment control trials. This study has shown that immune escape variants may be from those arising from CTL epitopes outside the HIV_gagconsv. However, functional and molecular studies on the nature and characteristics of HIV-1 strains in latent reservoirs need to be carried out to ascertain our claims. Furthermore, we cannot fully corroborate this theory in our study because of the relatively low sample size.

Also, universal HIV-1 vaccines are supposed to be broadly effective against all HIV-1 clades. However, immune escape strains encoding CTL epitopes outside the HIV_gagconsv may reduce the sensitivities of these vaccines. Observations of CTL escape mutants after seroconversion in this study suggest that immune pressures by other cells other than CTL may aid the generation of CTL immune escape mutants. Mapping HIV immune epitopes in different regions of the genome will further clarify this hypothesis (Matthews et al., 2012; Adland et al., 2013). As observed previously in other studies, CTL epitope KAFSPEVIPMF was the most conserved (Garcia-knight et al., 2016; Gama Caetano et al., 2018). This epitope has been associated with very low frequencies of CTL response selective pressures and has been a choice for many T cell-based HIV vaccines (Hanke, 2019). However, two substitutions previously associated with immune escape, K162R, and A163G were observed in this epitope for Subtype A during the early stages of infection.

On the other hand, CTL epitope DTINEEAAEWDR was associated with more substitutions, although most of these mutations reverted to wild type after seroconversion. This phenomenon was observable in both subtypes A and G. It seems that although more mutations were observed during the early stages of HIV infection, reversions of these mutations occurred later on in infection. Previous studies have also associated CTL epitopes’ reversions with the early stages of HIV infection (Li et al., 2007). This epitope was included in the second generation of functionally conserved HIV DNA vaccines because of its high conservation and coverage of strains diversity (Abdul-Jawad et al., 2016; Hanke, 2019). The immunogenicity of this epitope in the vaccine construct was also observed in macaques and untreated HIV infected individuals (Ondondo et al., 2016). These unique properties of this epitope were also observed in this study. However, a non-synonymous substitution, E203D, was observed in CTL epitope DTINEEAAEWDR. The selection of this epitope for immune escape strains has been previously described (Murakoshi et al., 2019). However, the substitution has also been shown not to impact the epitope’s immunogenicity as a vaccine construct (Ondondo et al., 2016). While the substitution may not affect function, it may be integrated into the latent reservoir landscape. Hence, the amino acid position should be excluded in the epitope’s design as a DNA vaccine construct.

CTL epitope TSTLQEQIR was conserved for subtype G. However, this epitope does not possess high coverage for subtype A sequences. Previous studies have documented that TSTLQEQIR may have lower HIV-1 strain diversity coverage, may be presented early, and probably associated with elite controllers (O’Connell et al., 2011; Balamurugan et al., 2013; Ondondo et al., 2016). This epitope in T cell-based HIV-1 DNA vaccines has shown moderate coverage but a strong HIV-1 specific CTL (Ondondo et al., 2016). The epitope may not be functional against subtype A and may probably be expressed by rare HLAs since it was identified in only six individuals in this study. This study does not favor using the epitope in a vaccine construct for the West African region where subtype A predominantly circulates. However, the epitope’s association with HIV-1 RNA viral load control and NEF gene downregulation (O’Connell et al., 2011; Balamurugan et al., 2013) is a plus for its use in a therapeutic HIV-1 DNA vaccine.

Several previously recognized immune escape substitutions were observed in this study. Majority of these substitutions emanated from epitopes outside the HIV_gagconsv. This is the first longitudinal study from West Africa on the kinetics of previously recognized functionally conserved epitopes of the HIV-1 GAG gene to the best of our knowledge. It is worthy of note that these epitopes have already been used in second-generation T cell HIV-1 DNA vaccines as a proof of concept. Our study provides real-life evidence of the immunodominance, conservative, and highly diverse strain coverage of these epitopes. These properties form the basis of the strategies employed in the design of conserved region vaccines.

We have also shown that numerous non-synonymous substitutions associated with CTL epitopes outside the HIV_gagconsv occur during the early stages of HIV-1 infection among HIV-1 subtypes and recombinant forms circulating in West Africa. It is essential to state that these substitutions were identified from HIV-1 DNA sequences against plasma RNA used in a similar study (Gounder et al., 2015). Proviral sequences have previously been associated with rare mutations on CTL epitopes (Gama Caetano et al., 2018; Lee et al., 2019). These substitutions may have to be considered in designing universal and therapeutic vaccines for HIV-1 strains circulating in West African countries (Ndung’u et al., 2019; Shu et al., 2020). Recent studies have shown the significant role of poorly recognized CTL epitopes in viral escape (Grossman et al., 2019).

While H28K, M30R, A224P, and A248G non-synonymous substitutions were observed before and after seroconversion, others, namely V82I and Y86W, were consistently identified after seroconversion. V82I has been previously identified with the emergence of higher viral loads in studies among HIV-infected individuals (Arcia et al., 2018; Karlsson et al., 2020). On the other hand, Y86W was associated with HIV-1 clade B and E (Fukada et al., 2002). Besides previous studies, we have also reported the high levels of creatinine among HIV-infected Africans during the early stages of infection(Bruggeman et al., 2000; Marras et al., 2002; Olusola et al., 2017, 2020b). In our study, we also showed that immune activation of CTL was ongoing and correlated positively with high creatinine levels in these early HIV-1 infected individuals (Olusola et al., 2020b). In this present study, we showed that a reduction in creatinine concentrations occurred after seroconversion. CTL immune pressures may be associated with high creatinine levels in Africans. However, this needs further investigation.

In summary, we have shown that there is a high genetic diversity of HIV-1 strains in Nigeria. Also, very high frequencies of non-synonymous substitutions occur in the HIV-1 GAG gene during the early stages of infection up until seroconversion. These substitutions include previously mapped CTL epitope immune escape mutants that are frequent in epitopes outside the HIV_gagconsv. Observation of the immunodominance of functionally conserved epitopes used in current T cell-based HIV-1 DNA vaccines in this study emphasizes the usefulness of these vaccines in a region where it is highly needed (Ndung’u et al., 2019). However, future directions for slight modifications to the use of the epitopes in the West African region are also noted. CTL immune pressure likely leaves different footprints and signature patterns on HIV-1 GAG epitopes within and outside the HIV_gagconsv.

## Data Availability Statement

The datasets presented in this study can be found in online repositories. The names of the repository/repositories and accession number(s) can be found in the article/Supplementary Material.

## Ethics Statement

The studies involving human participants were reviewed and approved. This research was conducted in accordance with the declaration of Helsinki. Experiments were conducted with the understanding and the consent of each participant. Ethical approvals for this research were obtained from the University of Ibadan/University College Hospital (UI/UCH) Research and Ethics Committee (UI/EC/15/0076) and the Oyo State Ministry of Health Committee on Human Research (AD13/479/951). All results were delinked from patient identifiers and anonymized. The patients/participants provided their written informed consent to participate in this study.

## Author Contributions

BO, DO, and GO conceptualized and designed the study. BO preformed the experiments, analysed and interpreted the data as well as wrote the first draft of manuscript. DO and GO supervised the work and reviewed the manuscript. All authors contributed to the article and approved the submitted version.

## Conflict of Interest

The authors declare that the research was conducted in the absence of any commercial or financial relationships that could be construed as a potential conflict of interest.

## Footnotes

1 https://www.hiv.lanl.gov

2 http://dbpartners.stanford.edu/RegaSubtyping/

3 http://comet.retrovirology.lu

4 http://www.ncbi.nlm.nih.gov/Blast.cgi

5 http://jphmm.gobics.de/submission_hiv

6 https://www.hiv.lanl.gov/content/sequence/NEWALIGN/align.html

7 http://www.hiv.lanl.gov/content/sequence/VESPA/vespa.html

8 https://www.hiv.lanl.gov/content/immunology/variants/ctl_variant.html
