# Supplementary material for: Non-synonymous Substitutions in HIV-1 GAG Are Frequent in Epitopes Outside the Functionally Conserved Regions and Associated With Subtype Differences
Source: Front Microbiol. 2021 Jan 11;11:615721. doi: 10.3389/fmicb.2020.615721 (PMC7829476; doi:10.3389/fmicb.2020.615721)
Supplement: Supplementary file 1 [file Table_1.DOCX]

Supplementary File 1

|  | **Before seroconversion** | |  |  |  |  |  | **After seroconversion** | |  |  |
| --- | --- | --- | --- | --- | --- | --- | --- | --- | --- | --- | --- |
| Substitution | MHR | without MHR | Subtype | Sample |  |  | Substitution | MHR | without MHR | Subtype | Sample |
| **H28K** |  | 1 | MN943614 | EHIV013 |  |  | **H28K** |  | 1 | MN943631 | EHIV013 |
| **M30R** |  | 1 | MN943614 |  |  |  | **M30R** |  | 1 | MN943631 |  |
| **L31M** |  | 1 | MN943630 | EHIV012 |  |  | **T72S** |  | 1 |  | EHIV013 |
| **G49S** |  | 1 | MN943614 | EHIV013 |  |  | **R76K** |  | 1 | MN943631 | EHIV013 |
| **S72T** |  | 1 | MN943613 |  |  |  | **V82I** |  | 2 | MN9433631 | EHIV013 |
| **T72S** |  | 1 |  | EHIV013 |  |  |  |  |  | MN943630 | EHIV012 |
| **L101V** |  | 2 | MN943626 | EHIV012 |  |  | **Y86W** |  | 2 | MN943630 | EHIV012 |
|  |  |  | MN943624 |  |  |  |  |  |  | MN943631 | EHIV013 |
| **N124S** |  | 1 | MN943614 | EHIV013 |  |  | **N124S** |  | 1 | MN943631 | EHIV013 |
| **I147L** |  | 1 | MN943614 |  |  |  | **S125*** |  | 1 | MN943631 | EHIV013 |
| **S172T** |  | 2 | MN943626 | EHIV012 |  |  | **N139R** |  | 1 | MN943631 | EHIV013 |
|  |  |  | MN943624 |  |  |  | **A146S** |  | 1 | MN943631 | EHIV013 |
| **I223V** |  | 1 | MN943614 | EHIV013 |  |  | **I147M** |  | 1 | MN943631 | EHIV013 |
| **A224P** |  | 1 | MN943614 | EHIV013 |  |  | **F172S** |  | 1 | MN943630 | EHIV012 |
| **A248G** |  | 1 | MN943614 | EHIV013 |  |  | **L215V** |  | 1 | MN943631 | EHIV013 |
| **G248R** |  | 2 | MN943626 | EHIV012 |  |  | **A223I** |  | 1 | MN943631 | EHIV013 |
|  |  |  | MN943624 |  |  |  | **A224P** |  | 1 | MN943631 | EHIV013 |
| **E203D** | 1 |  | MN943622 | EHIV022 |  |  | **A248G** |  | 1 | MN943631 | EHIV013 |
|  |  |  |  |  |  |  | **N252S** |  | 1 | MN943631 | EHIV013 |
|  |  |  |  |  |  |  | **D260E** |  | 1 | MN943631 | EHIV013 |
|  |  |  |  |  |  |  | **I267V** |  | 1 | MN943631 | EHIV013 |

| S/N | Mutation | LANL description of mutation type | Reference where mutation was described |
| --- | --- | --- | --- |
|  | H28K | Diminished response; subtype specific no susceptible form | Goulder et al., 1997; Phillips et al., 1991 |
|  | M30R | Escape; Replicative capacity reduced; observed variant | Goulder et al., 1997; Phillips et al., 1991; Zhao et al., 2007 |
|  | M30K | Subtype specific non susceptible form; diminished response | <https://www.hiv.lanl.gov/content/immunology/variants/ctl_variant.html> |
|  | L31M | Observed variant | <https://www.hiv.lanl.gov/content/immunology/variants/ctl_variant.html> |
|  | G49S | Subtype specific non susceptible form | <https://www.hiv.lanl.gov/content/immunology/variants/ctl_variant.html> |
|  | S72T | Observed variant | <https://www.hiv.lanl.gov/content/immunology/variants/ctl_variant.html> |
|  | G72S; T72S | Subtype specific non susceptible form | <https://www.hiv.lanl.gov/content/immunology/variants/ctl_variant.html> |
|  | R76K | Observed variant | <https://www.hiv.lanl.gov/content/immunology/variants/ctl_variant.html> |
|  | R76K | Decreased from earlier visit | Caetano et al., 2018 |
|  | V82L | Reversion in viral controllers | Caetano et al., 2018 |
|  | V82I | Immune escape mutation | Arcia et al., 2018; Tenner et al., 2009; Bennett et al., 2010; Goulder et al., 1997 |
|  | I82V | Observed variant | Goulder et al., 1997 |
|  | W86C | Observed variant | <https://www.hiv.lanl.gov/content/immunology/variants/ctl_variant.html> |
|  | Y86W | Reversion? | <https://www.hiv.lanl.gov/content/immunology/variants/ctl_variant.html> |
|  | R91K | Susceptible form | <https://www.hiv.lanl.gov/content/immunology/variants/ctl_variant.html> |
|  | L101V | Observed variant | <https://www.hiv.lanl.gov/content/immunology/variants/ctl_variant.html> |
|  | A118P | Reversions | Caetano et al., 2018 |
|  | D121A | Changes in viral controllers | Caetano et al., 2018 |
|  | N124K; N124S | Escape | <https://www.hiv.lanl.gov/content/immunology/variants/ctl_variant.html> |
|  | S125G | Escape | <https://www.hiv.lanl.gov/content/immunology/variants/ctl_variant.html> |
|  | S125R | Reversion | <https://www.hiv.lanl.gov/content/immunology/variants/ctl_variant.html> |
|  | S125* | Escape | <https://www.hiv.lanl.gov/content/immunology/variants/ctl_variant.html> |
|  | S126G | Observed variant; replicative capacity is not abrogated | <https://www.hiv.lanl.gov/content/immunology/variants/ctl_variant.html> |
|  | Q127* | Escape | Zhao et al., 2007 |
|  | N139R | Subtype specific non susceptible form | <https://www.hiv.lanl.gov/content/immunology/variants/ctl_variant.html> |
|  | A146P/A146S/I147L | Main alterations in viral controllers;  Mutations described in several studies as commonly arising in individuals carrying HLA-B*57 and B*58 alleles, despite resulting in loss of viral fitness.  Not here | Caetano et al., 2018; Feeney et al., 2005; Tang et al., 2010; Durand et al., 2010; Buckheit et al., 2012 |
|  | A146S | HLA association; Escape documented | <https://www.hiv.lanl.gov/content/immunology/variants/ctl_variant.html> |
|  | I147L | HLA association; Escape documented | <https://www.hiv.lanl.gov/content/immunology/variants/ctl_variant.html> |
|  | I147L | Reversion in viral controllers | Caetano et al., 2018 |
|  | I147M | HLA association; escape documented | <https://www.hiv.lanl.gov/content/immunology/variants/ctl_variant.html> |
|  | V158A | Susceptible form | <https://www.hiv.lanl.gov/content/immunology/variants/ctl_variant.html> |
|  | S172T | HLA associations; diminished response, Escape mutations | <https://www.hiv.lanl.gov/content/immunology/variants/ctl_variant.html> |
|  | F172S; F172T | Observed variant | Bukheit et al., 2012 |
|  | Q182G | Observed variant; Escape variant | Kloverpris et al., 2016 |
|  | L215V | Susceptible form | <https://www.hiv.lanl.gov/content/immunology/variants/ctl_variant.html> |
|  | H219A | Non susceptible forms | Hoof et al., 2010 |
|  | I223H; I223V | Observed variant | Hoof et al., 2010 |
|  | A223I | Diminished response | Hoof et al., 2010 |
|  | V215L | Naturally occurring susceptible form | Sispas et al., 1997 |
|  | V218A | Non susceptible form | Sispas et al., 1997; Hoof et al., 2010 |
|  | A224P | Non susceptible form | <https://www.hiv.lanl.gov/content/immunology/variants/ctl_variant.html> |
|  | A248T | Observed variant | Leslie et al., 2004 |
|  | A248G | Subtype specific non susceptible form; diminished response | Goulder et al., 2004 |
|  | G248R/G248A | Calculated Escape; diminished response | <https://www.hiv.lanl.gov/content/immunology/variants/ctl_variant.html> |
|  | N252G | Replicative capacity reduced | <https://www.hiv.lanl.gov/content/immunology/variants/ctl_variant.html> |
|  | N252S | Non susceptible form |  |
|  | D260E | Diminished HLA binding or increased off rate, diminished response, subtype specific non susceptible form | <https://www.hiv.lanl.gov/content/immunology/variants/ctl_variant.html> |
|  | K263R | Non susceptible form | Turnbull et al., 2006 |
|  | I267V | Diminished response; non susceptible form; susceptible form | <https://www.hiv.lanl.gov/content/immunology/variants/ctl_variant.html> |

**Description of terms**

**HLA association**: Variant is statistically associated with a particular HLA molecule. Since we focus on experimentally verified epitope variants, the variant with this mutation is entered only if it is already described as an experimentally determined mutation.

**Calculated Escape**: Predicted escape as shown by statistical correlation or other computational methods in a large cohort. Since we focus on experimentally verified epitope variants, the variant with this mutation is entered only if it is already described as an experimentally determined mutation.

**Compensatory Mutation**: Variant is associated with a compensatory mutation. This could be a compensatory mutation outside epitope boundaries, or a mutation within epitope boundaries, compensating for the same epitope or for a variant of another epitope. Each particular entry is explained in the variant note.

**Diminished response**: Experimental data suggests a partial escape by decreased CTL response, but authors do not call it an escape (judgment call of annotator).

**Escape documented in this paper**: Same as NSF (non-susceptible form) but called an escape when dynamic changes seen in a longitudinal or transmission study or when author claims escape.

**Non susceptible form**: No CTL response when patient cells are challenged with the variant peptide.

**Observed variant**: variant sequence observed in longitudinal or transmission study.

**Reversion**: Variant reverts to wild type epitope sequence as documented by sequence, experimental studies or literature.

**Replicative capacity is not abrogated**: Variant does not cause loss of viral replication, as shown by replication assays.

**Replicative capacity reduced**: Variant is associated with a reduction or loss (abrogation) of replication of the virus, as shown by replication assays.

**Susceptible form**: CTL response is elicited when patient cells are challenged with the variant peptide.

**Subtype specific non susceptible form**: No CTL response when patient cells are challenged with the variant peptide in the course of subtype comparative studies, however the same epitope from a different subtype does elicit CTL response.

**Subtype specific susceptible form**: CTL response is elicited when patient cells are challenged with the variant peptide in the course of subtype comparative studies, i.e. patient cells can recognize at least two different viral subtype variants.
